# Travel Time as an Indicator of Poor Access to Care in Surgical Emergencies

**DOI:** 10.1001/jamanetworkopen.2024.55258

**Published:** 2025-01-21

**Authors:** Nina M. Clark, Alexandra H. Hernandez, Mia S. Bertalan, Virginia Wang, Sarah L. M. Greenberg, Andrew M. Ibrahim, Barclay T. Stewart, John W. Scott

**Affiliations:** 1 Department of Surgery, University of Washington, Seattle; 2 Division of Pediatric General and Thoracic Surgery, Seattle Children’s Hospital, Seattle, Washington; 3 Department of Surgery, University of Michigan, Ann Arbor; 4 Center for Healthcare Outcomes and Policy, Institute for Healthcare Policy and Innovation, University of Michigan, Ann Arbor; 5 Visual Abstract Editor, JAMA Network, Chicago, Illinois; 6 Taubman College of Architecture and Urban Planning, University of Michigan, Ann Arbor; 7 Division of Trauma, Burn, and Critical Care Surgery, University of Washington, Seattle; 8 Institute for Health Metrics and Evaluation, University of Washington, Seattle

## Abstract

**Importance:**

Timely access to care is a key metric for health care systems and is particularly important in conditions that acutely worsen with delays in care, including surgical emergencies. However, the association between travel time to emergency care and risk for complex presentation is poorly understood.

**Objective:**

To evaluate the impact of travel time on disease complexity at presentation among people with emergency general surgery conditions and to evaluate whether travel time was associated with clinical outcomes and measures of increased health resource utilization.

**Design, Setting, and Participants:**

This retrospective cohort study used administrative statewide inpatient and emergency department databases with linkage across encounters, including nearly every inpatient or emergency department encounter in the states of Florida and California in 2021. Participants included adult patients who presented to an emergency department with 1 of 5 common emergency surgical conditions. Data were collected from January to December 2021 and analyzed from June to December 2023.

**Exposure:**

The primary exposure was travel time from the patient’s home to the facility where they initially received emergency care.

**Main Outcomes and Measures:**

The primary outcome of interest was surgical disease complexity at the time of presentation to emergency care. Secondary outcomes included inpatient complications, mortality, and indicators of health system resource utilization. Multivariable logistic regression models were used, and adjusted odds ratios (aOR) and 95% CIs were reported.

**Results:**

Among 190 311 adults with emergency general surgery conditions, 7138 (3.8%) lived further than 60 minutes from the facility where they sought emergency care. Longer travel times were associated with higher odds of complex disease presentation for travel time of more than 120 minutes vs 15 minutes or less (aOR, 1.28; 95% CI, 1.17-1.40). Patients with a travel time 60 minutes or more were more likely to require operative intervention (aOR, 1.17; 95% CI, 1.10-1.26), inpatient admission (aOR, 1.41; 95% CI, 1.33-1.50), interfacility transfer (aOR, 1.32; 95% CI, 1.15-1.51), and longer inpatient stay (adjusted mean difference, 0.47 days; 95% CI, 0.35-0.59), and had higher charges (adjusted mean difference, $8284; 95% CI, $5532-$11 035).

**Conclusions and Relevance:**

In this cohort study of patients with emergency surgical conditions, travel time to emergency care was associated with markers of delayed presentation and increased facility resource utilization. As opposed to static measures, such as rurality, travel time may serve as a more useful metric to inform policy efforts aimed at preserving access to care amidst rural hospital closures and regionalization.

## Key Points

**Question:**

How does proximity to emergency care impact disease complexity at presentation for patients with acute surgical conditions?

**Findings:**

In this cohort study of 190 311 patients, those with longer travel times had higher odds of complex surgical disease at presentation. Patients with a travel time of 60 minutes or longer were more likely to have higher charges and require operative intervention, transfer, inpatient admission, and longer inpatient stay.

**Meaning:**

These findings suggest that policymakers should account for proximity to emergency care when considering impacts of rural hospital closures and policy efforts to preserve timely access to care.

## Teaser

This cohort study examines patient travel time to hospitals for surgical emergencies and assesses disease complexity at presentation.

## Introduction

Timely access to care is a critical component of health care delivery and a key quality indicator. Acute surgical conditions, which account for nearly 10% of annual hospitalizations in the US, are commonly used as a benchmark for emergency service availability, as delays in care can increase the likelihood of complex disease at presentation (eg, appendicitis with perforation or abscess).^1,2,3,4,5,6^ The World Bank recommends using travel time as a proxy for timely access to care in these surgical emergencies.^7,8,9^ Despite this, little data guide the application of this metric among emergency surgical patients in the US.^10^ As regionalization continues to impact service delivery and policy efforts to promote access to emergency care are instituted at the federal level,^11^ robust metrics for timely access to care and its downstream consequences are needed.

However, the association between travel time to emergency care and delayed presentation for acute surgical conditions is poorly understood. Prior studies have demonstrated that patients with barriers to timely access to care and inadequate financial risk protection may delay presentation for surgical conditions and are at increased likelihood for presenting with higher complexity disease.^2,3,4,12,13,14,15^ Disease complexity at presentation for patients with emergency surgical conditions is thus a potential metric for objectively evaluating access to care,^1,16^ but has not yet been studied in relation to travel time to health care facilities. By understanding the association between proximity to surgical care and timely presentation in surgical emergencies, policymakers, and health care leaders can better assess the impacts of hospital closures on patients and the systems that serve them.

In this context, we sought to identify the association between travel time to emergency care and delayed presentation in surgical emergencies. Our primary aim was to evaluate the impact of travel time on disease complexity at presentation among people with emergency general surgery conditions. Our secondary aim was to evaluate whether travel time was associated with clinical outcomes and measures of increased health care resource utilization.

## Methods

### Data Source

The Strengthening the Reporting of Observational Studies in Epidemiology (STROBE) reporting guideline were followed.^17^ This cohort study was approved by the institutional review board of the University of Washington. Informed consent was not required because patient data were deidentified.

We used data from the 2021 Healthcare Cost and Utilization Project (HCUP) State Inpatient and Emergency Department (ED) Databases from Florida and California. These include information for approximately 97% of hospital discharges in each state, incorporating data about all encounters regardless of payer type. Patients discharged or transferred from an ED encounter are included in the state ED database; those who are admitted from the ED are only included in the state inpatient database, allowing for analyses of nearly all emergency health care encounters for a state within a given year, regardless of ED disposition.

### Study Cohort and Covariates

We included encounter data from all adult patients (aged 18 years or older) who had any of 5 emergency general surgery conditions at the time of hospital or ED presentation, including appendicitis, cholecystitis, diverticulitis, hernia, and bowel obstruction. These conditions have been used to identify delays in access to emergency general surgery care in previous studies.^2,13,15^ We used the *International Statistical Classification of Diseases and Related Health Problems, Tenth Revision *(*ICD-10*) codes to identify patients meeting diagnostic criteria for inclusion in the emergency general surgery cohort.^1,18^ The American Association for the Surgery of Trauma disease grading scale for each condition was used to define disease complexity at presentation by identifying *ICD-10* codes corresponding to higher and lower complexity disease.^1,18,19^ Only patients with a home zip code and hospital information available were included. We used the home and hospital zip codes provided in HCUP to exclude patients who did not seek care in the same state as their home address to reduce misclassification of travel time due to patients who were traveling outside of their home state.

Sociodemographic and clinical characteristics included age (18 to 39 years, 40 to 64 years, 65 to 79 years, and 80 years or older), sex, race and ethnicity, insurance payer type (private for patients younger than age 65 years and 65 years or older, Medicare, Medicaid, other, uninsured), and median household income quartile for the patient’s zip code. Race and ethnicity data in HCUP are adopted from self-reported patient data recorded by hospitals. Race and ethnicity were included as potential confounders that may influence access to health care. For the purpose of this study, we categorized these into Hispanic, non-Hispanic Black, non-Hispanic White, and non-Hispanic other (including patients reporting non-Hispanic ethnicity and any racial identification other than White or Black) to allow for analysis.^20^ Medical comorbidities were aggregated into the Elixhauser comorbidity index and subsequently categorized (less than 0, 0, 1 to 5, 6 to 13, and 14 or more).^21,22^

### Exposure

Our primary exposure was the travel time in minutes from the patient’s home to the hospital where they first sought emergency care, regardless of whether those facilities provided definitive surgical care or transferred the patient. This was intentional to evaluate disease complexity at the time of initial presentation as a proxy for access to care. HCUP provides patient home zip codes; therefore, population-weighted centroids of each zip code were used as the origin location. The use of zip code population centroids may be a more accurate method to estimate travel time compared with geometry-based methods and has been validated in administrative databases.^23,24^ Hospital details, including addresses, were obtained via linkage of the American Hospital Association Annual Survey database with HCUP data. We used the georoute package in Stata version 18.0 (StataCorp) to obtain travel time in minutes required to drive by car between these 2 points under normal traffic conditions.^25^ Travel time was then categorized into 15 minutes or less, 16 to 30 minutes, 31 to 60 minutes, 61 to 120 minutes, and more than 120 minutes for our primary analysis and into a binary of less than 60 minutes and 60 minutes or more in our secondary analyses.^26,27^ Rurality of the patient’s home address was defined by linking each patient’s home zip code to its corresponding Rural-Urban Commuting Area (RUCA) code. A RUCA code of 3 or more was considered metropolitan, 4 to 6 was considered micropolitan, and more than 6 was considered rural.

### Primary Outcome

Our primary outcome was the complexity of surgical disease at presentation. We defined this using previously published *ICD-10* codes that have been used to clinically validate the disease grading system published by the American Association for the Surgery of Trauma.^1,16^ Patients were categorized as having higher or lower complexity emergency general surgery disease at the time of presentation through the use of *ICD-10* codes, only including conditions that were flagged as being present on admission in HCUP.

### Secondary Outcomes

We also evaluated whether prolonged travel time to emergency care was associated with clinical and health care resource utilization outcomes. Mortality was defined as health care encounters associated with death or discharge to hospice and was subsequently categorized as either emergency department or inpatient mortality based on encounter type. The development of any in-hospital complication was identified using *ICD-10 *codes that were not present on admission.^2,15^ Operative intervention was defined as an encounter during which any emergency general surgery–related surgical procedure was performed, using *ICD-10-Procedure Codes*.^15^ We defined inpatient admission as those encounters included in only the state inpatient database. Interfacility transfers were identified in HCUP using previously published methods that leveraged the VisitLink variable.^28^ Finally, we evaluated total charges and total length of stay for each encounter. When transfers occurred, outcomes were considered across the entire episode of care (including both the pretransfer and posttransfer encounters in a transfer episode).

### Statistical Analyses

To determine associations between travel time, rurality, and surgical disease complexity at presentation, we used multivariable logistic regression models with robust standard errors. We report estimated adjusted odds ratios (aORs) and 95% CIs, adjusted for age, sex, race and ethnicity, insurance payer type, income quartile, Elixhauser comorbidity, and primary surgical condition as defined previously. Models included both rurality and travel time related to patients’ home zip codes. The same covariables were used to construct multivariable logistic or linear models for each of the secondary outcomes. *P *< .05 was considered statistically significant. All statistical analyses were performed using STATA version 18.0 (StataCorp), with data visualization using R version 4.3.2 (R Project for Statistical Computing). Data were collected from January to December 2021 and analyzed from June to December 2023.

We performed several sensitivity analyses to ensure that our findings were not related to model specification. First, we used multiple cutoffs for our binary time to care exposure measure, including 90 minutes and 120 minutes. We then stratified analyses by patient rurality into rural vs micropolitan and metropolitan populations and repeated analyses among those subpopulations. We repeated analyses including all patients, not only those who sought emergency care within their own state. Finally, given the low proportion of patients with cholecystitis and bowel obstruction who presented with higher-complexity disease, we repeated our analyses among patients with appendicitis, diverticulitis, and hernia.

## Results

### Sociodemographic and Clinical Characteristics

Data from 190 311 patients were analyzed. Of these patients, 78 710 were aged 40 to 64 years (41.4%), 100 627 were female (52.9%), 59 069 were Hispanic patients (31.3%), 96 709 were non-Hispanic White patients (51.2%), 183 173 patients (96.2%) lived within 60 minutes of the hospital where they sought emergency care, and 7138 patients (3.8%) had travel times of 60 minutes or more (Table 1). Sociodemographic and clinical characteristics were similar between patients who traveled less than 60 minutes for care vs those traveling for 60 minutes or longer (Table 1), and across narrower travel time categories (eg, 15 minutes or less, 16 to 30 minutes, 30 to 60 minutes, 60 to 120 minutes, more than 120 minutes) (eTable 1 in Supplement 1).

**Table 1.** Sociodemographic and Clinical Characteristics by Travel Time

Characteristic	Patients, No. (%)		
	Total	Travel time	
		<60 min	≥60 min
No.	190 311 (100.0)	183 173 (96.2)	7138 (3.8)
Age, y			
18-39	38 176 (20.1)	36 645 (20.0)	1531 (21.4)
40-64	78 710 (41.4)	75 593 (41.3)	3117 (43.7)
65-79	48 722 (25.6)	46 968 (25.6)	1754 (24.6)
≥80	24 703 (13.0)	23 967 (13.1)	736 (10.3)
Sex			
Male	89 684 (47.1)	86 100 (47.0)	3584 (50.2)
Female	100 627 (52.9)	97 073 (53.0)	3554 (49.8)
Race and ethnicity			
Hispanic	59 069 (31.3)	57 139 (31.4)	1930 (27.4)
Non-Hispanic			
Black	15 249 (8.1)	14 646 (8.1)	603 (8.6)
White	96 709 (51.2)	92 779 (51.1)	3930 (55.8)
Other^a^	17 705 (9.4)	17 121 (9.4)	584 (8.3)
Payer			
Medicare	74 430 (39.1)	71 854 (39.2)	2576 (36.1)
Medicaid	38 073 (20.0)	36 813 (20.1)	1260 (17.7)
Private, age, y			
<65	59 907 (31.5)	57 487 (31.4)	2420 (33.9)
≥65	5223 (2.7)	4987 (2.7)	236 (3.3)
Other	4655 (2.4)	4458 (2.4)	197 (2.8)
Uninsured	7969 (4.2)	7523 (4.1)	446 (6.3)
Median income quartile			
First (highest)	50 871 (26.9)	48 920 (27.0)	1951 (27.7)
Second	40 310 (21.4)	38 829 (21.4)	1481 (21)
Third	45 344 (24.1)	49 474 (24.0)	1870 (26.6)
Fourth (lowest)	51 933 (27.6)	50 252 (27.7)	1741 (24.7)
Elixhauser Comorbidity Index			
<0	53 212 (28.0)	51 275 (28.0)	1937 (27.1)
0	85 686 (45.0)	82 415 (45.0)	3271 (45.8)
1-5	23 160 (12.2)	22 360 (12.2)	800 (11.2)
6-13	10 972 (5.8)	10 556 (5.8)	416 (5.8)
≥14	17 281 (9.1)	16 567 (9.0)	714 (10.0)
Primary surgical condition			
Appendicitis	29 002 (15.2)	27 781 (15.2)	1221 (17.1)
Cholecystitis	35 462 (18.6)	34 152 (18.6)	1310 (18.4)
Hernia	37 931 (19.9)	36 475 (19.9)	1456 (20.4)
Intestinal obstruction	47 680 (25.1)	45 825 (25.0)	1855 (26.0)
Diverticulitis	40 236 (21.1)	38 940 (21.3)	1296 (18.2)
Rurality			
Metropolitan	179 399 (94.3)	173 400 (94.7)	5999 (84.0)
Micropolitan	7232 (3.8)	6757 (3.7)	475 (6.7)
Rural	3679 (1.9)	3015 (1.6)	664 (9.3)

^a^ Non-Hispanic other includes patients reporting non-Hispanic ethnicity and any racial identification other than White or Black.

### Higher-Complexity Emergency General Surgery Presentation by Travel Time

**Figure 1.**
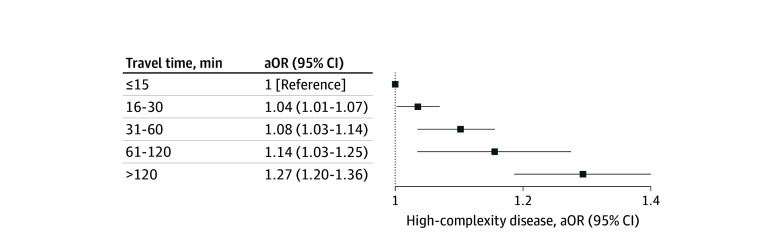
Likelihood of Higher-Complexity Surgical Disease by Travel Time aOR indicates adjusted odds ratio.

After adjusting for sociodemographic and clinical characteristics, including rurality, travel time was associated with increased odds of higher complexity disease at presentation (Table 2). Additionally, 38 073 patients (20.0%) were Medicaid-insured and 7969 patients (4.2%) lacked insurance coverage. For example, 18 771 of 106 726 of patients (17.6%) who had travel times of 15 minutes or less compared with 830 of 3921 patients (21.2%) who had travel times longer than 120 minutes (aOR, 1.28 [95% CI, 1.17-1.40]) were more likely to present with a higher-complexity condition. Ordinal groups of travel time (15 minutes or less, 16 to 30 minutes, 31 to 60 minutes, 61 to 120 minutes, longer than 120 minutes) had a monotonic increase in odds of higher complexity surgical disease at every interval (Figure 1). Similar trends were observed when we included patients who presented to a facility outside their home state (eTable 2 in Supplement 1). Unlike travel time, rurality was not associated with increased disease complexity at presentation in our risk-adjusted models; rather, patients from rural areas were less likely to present with complex disease once travel time was accounted for (aOR, 0.83; 95% CI, 0.75-0.92).

**Table 2.** Multivariable Logistic Regression, Odds of Higher-Complexity Surgical Disease at Presentation

Characteristic	Total patients, No.	Higher-complexity disease, No. (%)	aOR (95% CI)
Sociodemographic characteristic			
Age, y			
≥80	24 703	4095 (16.6)	1 [Reference]
65-79	48 722	9018 (18.5)	0.55 (0.51-0.60)
40-64	48 710	14 928 (30.6)	0.76 (0.72-0.82)
18-39	38 176	6079 (15.9)	1.06 (1.02-1.12)
Sex			
Male	89 684	17 648 (19.7)	1 [Reference]
Female	100 627	16 472 (16.4)	0.88 (0.85-0.90)
Race			
Hispanic	59 069	9497 (16.1)	0.85 (0.82-0.88)
Non-Hispanic			
Black	15 249	2578 (16.9)	0.85 (0.80-0.89)
White	96 709	18 968 (19.6)	1 [Reference]
Other^a^	17 705	2817 (15.9)	0.90 (0.85-0.94)
Payer			
Medicaid	38 073	5803 (15.2)	1 [Reference]
Medicare	74 430	13 125 (17.6)	0.98 (0.92-1.04)
Private (<65 y old)	59 907	11 701 (19.5)	1.29 (1.22-1.37)
Private (≥65 y)	5223	926 (17.7)	0.94 (0.86-1.02)
Other	4655	828 (17.8)	1.01 (0.92-1.11)
Uninsured	7969	1725 (21.6)	1.34 (1.24-1.45)
Median income quartile			
First (highest)	51 993	9077 (17.5)	1 [Reference]
Second	40 310	7248 (18.0)	1.10 (1.06-1.14)
Third	45 344	8258 (18.2)	1.12 (1.08-1.16)
Fourth (lowest)	50 871	9186 (18.1)	1.01 (0.97-1.05)
Clinical characteristic			
Elixhauser Comorbidity Index			
<0	53 212	11 500 (21.6)	1 [Reference]
0	85 686	12 801 (14.9)	0.38 (0.37-0.39)
1-5	23 160	4068 (17.6)	0.67 (0.64-0.70)
6-13	10 972	2196 (20.0)	1.18 (1.11-1.26)
≥14	17 281	3555 (20.6)	1.34 (1.27-1.41)
Primary surgical condition			
Appendicitis	29 002	10 793 (37.2)	1 [Reference]
Cholecystitis	35 462	67 (0.2)	0.002 (0.002-0.003)
Hernia	37 931	12 050 (31.8)	0.67 (0.64-0.69)
Intestinal obstruction	47 680	1604 (3.4)	0.03 (0.03-0.04)
Diverticulitis	40 236	9606 (23.9)	0.43 (0.42-0.45)
Rurality measures			
Rurality			
Metropolitan	179 399	32 209 (17.9)	1 [Reference]
Micropolitan	7232	1276 (17.6)	0.92 (0.86-0.99)
Rural	3679	635 (17.3)	0.83 (0.75-0.92)
Travel time, min			
≤15	106 726	18 771 (17.6)	1 [Reference]
16-30	60 874	10 958 (18.0)	1.03 (1.002-1.06)
31-60	15 573	2953 (19.0)	1.09 (1.04-1.15)
61-120	3217	608 (18.9)	1.14 (1.03-1.26)
>120	3921	830 (21.2)	1.28 (1.17-1.40)

Abbreviation: aOR, adjusted odds ratio.

^a^ Non-Hispanic other includes patients reporting non-Hispanic ethnicity and any racial identification other than White or Black.

### Secondary Analyses: Clinical Outcomes and Health Care Resource Utilization

Patients with travel time longer than 60 minutes had 19% (95% CI, 12%-28%) higher odds of presentation with higher-complexity emergency general surgery disease, 32% (95% CI, 15%-51%) higher odds of interfacility transfer, 17% (95% CI, 10%-26%) higher odds of surgical intervention, and 41% (95% CI, 33%-50%) higher odds of hospital admission. Patients with prolonged travel times had overall longer length of stays (adjusted mean difference, 0.47 days; 95% CI, 0.35-0.59 days) and higher total charges (adjusted mean difference, $8284; 95% CI, $5532-$11 035) compared with patients with less than 60 minutes of travel time (Table 3). These findings were similar when patients were stratified by travel time longer than 90 minutes and 120 minutes (eTables 3 and 4 in Supplement 1).

**Table 3.** Associations Between Secondary Outcomes With a Travel Time of 60 Minutes or Longer

Secondary outcome	Travel time, No. (%)		aOR (95% CI)^a^
	≥60 min	<60 min	
Binary outcomes			
Complex disease at presentation	1438 (20.1)	32 682 (17.8)	1.19 (1.12-1.28)
Interfacility transfer	261 (3.7)	4270 (2.3)	1.32 (1.15-1.51)
Surgical intervention	3201 (44.8)	75 848 (41.4)	1.17 (1.10-1.26)
Hospital admission	4936 (69.2)	116 120 (63.4)	1.41 (1.33-1.50)
Inpatient mortality or complications^b^	294 (6.0)	6849 (5.9)	1.00 (0.88-1.14)
Continuous outcomes, mean (SD)			
Length of stay, d	3.1 (5.5)	2.7 (4.9)	0.47 (0.35-0.59)^c^
Total charges, USD	72 903 (118 088)	64 433 (91 674)	8284 (5532-11 035)^c^

Abbreviation: aOR, adjusted odds ratio.

^a^ aORs and adjusted differences: after adjustment for age, sex, race and ethnicity, payor type, income, comorbidity, primary emergency general surgery diagnosis, and rurality.

^b^ Mortality or complications outcome evaluated among patients who were admitted as inpatient.

^c^ Adjusted difference (95% CI).

### Subpopulation Analyses

**Figure 2.**
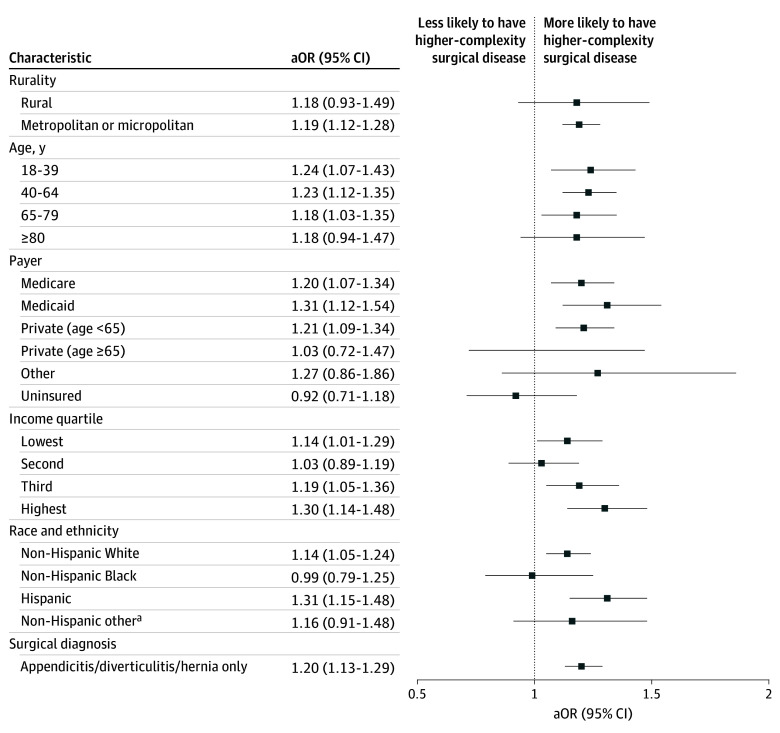
Likelihood of Higher-Complexity Surgical Disease at Presentation if Travel Time is 60 Minutes or Longer Among Subpopulations Adjusted odds ratios (aOR) presented after adjustment for primary surgical disease. ^a^Non-Hispanic other includes patients reporting non-Hispanic ethnicity and any racial identification other than White or Black.

Among subpopulations based on rurality, age, payer, income quartile, and race and ethnicity; and among a subpopulation of patients with appendicitis, diverticulitis, or hernia; those with travel time longer than 60 minutes were more likely to present with higher complexity surgical disease (Figure 2). While we were underpowered to detect statistical significance in several subpopulations, the odds of complex disease at presentation were numerically greater if travel times were 60 minutes or longer across almost every subpopulation.

## Discussion

In this study, patients with common acute surgical conditions who had prolonged travel time to emergency care were more likely to present with higher complexity disease, suggesting barriers to timely navigation of the health system. Longer travel times were also associated with greater health care resource utilization. These findings suggest that travel time may be a useful surrogate when assessing population-level access to emergency care, which could inform policies addressing timely access to care in at-risk groups.

Our findings suggest that prolonged travel time was associated with barriers to seeking and reaching care in surgical emergencies. We found a monotonic association between travel time and odds of higher complexity disease at presentation, which persisted even after stratification into sociodemographic subpopulations. Travel time has been used for decades in the global health literature as a measure of health care accessibility,^6,8,29^ and has been proposed as a potential metric in the US context.^9^ However, prior studies evaluating travel time to care in surgical disease have had 2 major limitations. First, they have predominantly evaluated smaller geographic regions, limiting applicability to the unique geographic and health care landscape of the US.^30^ Second, they have predominantly evaluated associations between travel time and mortality from surgical disease. We evaluated delayed presentations in surgical emergencies rather than downstream outcomes to more explicitly assess the impact of timely access to care. Patients with other clinical, financial, or systematic barriers to receiving surgical care were more likely to present with higher complexity surgical disease.^2,4,13,14,15,31^ Collectively, our study suggests that timeliness should be considered alongside affordability and equity as essential components of health care access. Importantly, we evaluated travel time from patients’ homes to the facilities where they received emergency care—regardless of whether those facilities were able to provide definitive surgical care. Therefore, we highlighted the potential benefit of preserving timely access to emergency departments that can provide initial care and stabilization of surgical patients, even if these facilities lack definitive surgical capabilities.

Prolonged travel time was not only associated with poor patient-level access to care, but with increased health system resource utilization as well. Patients with longer travel times were more likely to require an operation, be admitted as inpatients, undergo interfacility transfer, have longer lengths of stay, and incur higher charges. Prior studies have shown that patients living in rural areas utilized more health care resources,^32,33^ but these studies have not accounted for hospital proximity. We extended these findings to show that independent of rurality, travel time was associated with increased resource utilization in emergency surgical patients. Rurality is a static measure that does not necessarily reflect the accessibility of health care. For example, many rural hospitals are both accessible in a timely manner and capable of providing high-quality care to their communities.^34^ Using timeliness as a measure for access may therefore identify communities at risk for delayed presentation for emergency care that are masked by rural or urban classifications alone. If patients with prolonged travel times to care present with more complex diseases requiring access to more clinical resources, improving timely access to care could lower the burden on health care systems caring for these patients.

Our study provides 2 major insights to inform ongoing policy debates at the state and federal levels. First, this study highlights travel time to a hospital or emergency department as a valuable proxy measure for access to care at a population level. This may inform ongoing policy discussions around rural hospital closures and recent legislation aimed at preventing them. While openings or closures of rural hospitals may have substantial impacts on the timeliness of emergency care in the surrounding communities, they will not change the rurality of those communities. It may therefore be more useful for policymakers to understand the number of individuals living within 60 minutes of a hospital, as opposed to the number of patients who live in a zip code classified as rural. Considering geospatial proximity as a key metric of accessible care will align national efforts with global health policy recommendations from the World Bank and others. A second policy-relevant finding is the impact that greater travel time may have on health system utilization. The 2021 Consolidated Appropriations Act^11^ established the Rural Emergency Hospital designation, which allows eligible rural facilities to close their inpatient units in favor of providing emergency and outpatient services and receive additional federal funding and public payor reimbursement rates. To date, 21 hospitals have adopted Rural Emergency Hospital status.^35^ While our data support that access to emergency care may facilitate better outcomes and earlier presentation among emergency general surgery patients, the downstream effects on surgical transfers, access to operative and inpatient care, and capacity at tertiary referral facilities remain major points of concern.^36^ Future work must account for potential changes in how patients access and experience care in surgical emergencies as more facilities convert to Rural Emergency Hospitals. Surgical care may also be impacted by the expansion of quality programs, including the Emergency General Surgery Verification Program that was recently established by the American College of Surgeons.^37^ Our findings suggest that the geospatial distribution of quality emergency care may be critical to ensuring better surgical outcomes for all people in the US.

### Limitations

This study has limitations. First, HCUP data are based on hospital claims, and our primary outcome was dependent on *ICD-10* diagnosis coding; therefore, our data may be subject to misclassification due to inaccuracies in coding or differential access to diagnostics in different care settings. We attempted to address this by using clinically validated coding schemata that has used national data to measure various degrees of disease severity using *ICD-10* diagnosis codes.^1^ Second, although our analysis included 2 large, geographically and culturally diverse states, further investigation is necessary to determine whether similar patterns exist nationwide, particularly when evaluating the policy relevance of these findings. Our primary exposure was driving travel time under usual traffic conditions. While this is a common metric for geospatial access to care in global health literature,^38,39,40^ it does not account for differences in available modes of transportation that are available to individuals. Future studies would benefit from evaluating the intersection of geospatial distance to surgical care and transportation difficulties, which are a known barrier to timely access to care.^41,42^ Finally, while our study specifically aimed to evaluate timely access to emergency care, we were agnostic as to whether index facilities were the closest facility to the patient’s home, and capable of providing definitive surgical care for the patients’ condition. While some work has suggested that patients lacking geospatial access to care may choose to bypass the closest facilities,^43^ our results represent a pragmatic assessment of where patients received emergency care regardless of preference or proximity to other facilities and may better represent the current status of emergency care. However, further investigation into hospital bypass behavior among emergency general surgery patients and the accessibility of definitive surgical care among these populations is necessary to develop systems that ensure access for all patients.

## Conclusions

In this cohort study of adults with acute surgical conditions, those with prolonged travel time to emergency care were more likely to present with higher complexity disease, a marker for delays in receiving care. Prolonged travel time was associated with increased health care resource utilization at the system level in the form of longer hospital stays, more operative intervention, more transfers, and higher charges. These findings were robust to stratification across several key sociodemographic subpopulations. As policymakers work to preserve access to care in rural communities, using metrics that reflect barriers to reaching care and their downstream effect on resources will be key to developing interventions that ensure timely access to care in emergency situations.

## Notes

Supplement 1. **eTable 1.** Sociodemographic Characteristics According to Narrow Travel Time Categories **eTable 2.** Association Between Primary Outcome and Travel Time Categories, Including Patients Who Presented to a Hospital Outside of Their Home State **eTable 3.** Association Between Secondary Outcomes and Travel Time Greater Than 90 Minutes **eTable 4.** Association Between Secondary Outcomes and Travel Time Greater Than 120 Minutes

Supplement 2.
Data Sharing Statement
