# Supplementary material for: Travel Time as an Indicator of Poor Access to Care in Surgical Emergencies
Source: JAMA Netw Open. 2025 Jan 21;8(1):e2455258. doi: 10.1001/jamanetworkopen.2024.55258 (PMC11751744; doi:10.1001/jamanetworkopen.2024.55258)
Supplement: Supplement 1. — eTable 1. Sociodemographic Characteristics According to Narrow Travel Time Categories eTable 2. Association Between Primary Outcome and Travel Time Categories, Including Patients Who Presented to a Hospital Outside of Their Home State eTable 3. Association Between Secondary Outcomes and Travel Time Greater Than 90 Minutes eTable 4. Association Between Secondary Outcomes and Travel Time Greater Than 120 Minutes [file jamanetwopen-e2455258-s001.pdf]

# Supplemental Online Content

Clark NM, Hernandex AH, Bertalan MS, et al. Travel time as an indicator of poor access to care in surgical emergencies. *JAMA Netw Open*. 2025;8(1):e2455258. doi:10.1001/jamanetworkopen.2024.55258

**eTable 1.** Sociodemographic Characteristics According to Narrow Travel Time Categories

**eTable 2.** Association Between Primary Outcome and Travel Time Categories, Including Patients Who Presented to a Hospital Outside of Their Home State

**eTable 3.** Association Between Secondary Outcomes and Travel Time Greater Than 90 Minutes

**eTable 4.** Association Between Secondary Outcomes and Travel Time Greater Than 120 Minutes

This supplemental material has been provided by the authors to give readers additional information about their work.

© 2025 Clark NM et al. *JAMA Network Open*.

## eTable 1. Sociodemographic Characteristics According to Narrow Travel Time Categories

| | ≤15 minutes | 16-30 minutes | 31-60 minutes | 61-120 minutes | >120 minutes | Total |
|---|---|---|---|---|---|---|
| | N (%) | N (%) | N (%) | N (%) | N (%) | % |
| N | 106,726 (56.1%) | 60,874 (32.0%) | 15,573 (8.2%) | 3,217 (1.7%) | 3,921 (2.1%) | 190,311 |
| Age (years) | | | | | | |
| 18-39 | 21,296 (20.0%) | 12,054 (19.8%) | 3,295 (21.2%) | 698 (21.7%) | 833 (21.2%) | 38,176 |
| 40-64 | 42,994 (40.3%) | 25,851 (42.5%) | 6,748 (43.3%) | 1,419 (44.1%) | 1,698 (43.3%) | 78,710 |
| 65-79 | 27,331 (25.6%) | 15,680 (25.8%) | 3,957 (25.4%) | 824 (25.6%) | 930 (23.7%) | 48,722 |
| ≥80 | 15,105 (14.2%) | 7,289 (12.0%) | 1,573 (10.1%) | 276 (8.6%) | 460 (11.7%) | 24,703 |
| Sex | | | | | | |
| Male | 50,261 (47.1%) | 28,259 (46.4%) | 7,580 (48.7%) | 1,633 (50.8%) | 1,951 (49.8%) | 89,684 |
| Female | 56,465 (52.9%) | 32,615 (53.6%) | 7,993 (51.3%) | 1,584 (49.2%) | 1,970 (50.2%) | 100,627 |
| Race and Ethnicity | | | | | | |
| Non-Hispanic White | 53,588 (50.6%) | 30,479 (50.5%) | 8,712 (56.5%) | 1,836 (57.9%) | 2,094 (54.0%) | 96,709 |
| Non-Hispanic Black | 8,500 (8.0%) | 5,018 (8.3%) | 1,128 (7.3%) | 249 (7.8%) | 354 (9.1%) | 15,249 |
| Hispanic | 33,623 (31.7%) | 19,027 (31.5%) | 4,489 (29.1%) | 826 (26.0%) | 1,104 (28.5%) | 59,069 |
| Non-Hispanic Other | 10,203 (9.6%) | 5,826 (9.7%) | 1,092 (7.1%) | 261 (8.2%) | 323 (8.3%) | 17,705 |
| Payer | | | | | | |
| Medicare | 42,816 (40.1%) | 23,329 (38.3%) | 5,709 (36.7%) | 1,180 (36.7%) | 1,396 (35.6%) | 74,430 |
| Medicaid | 23,343 (21.9%) | 10,799 (17.7%) | 2,671 (17.2%) | 628 (19.5%) | 632 (16.1%) | 38,073 |
| Private (<65 years old) | 30,871 (28.9%) | 21,101 (34.7%) | 5,515 (35.4%) | 1,049 (32.6%) | 1,371 (35.0%) | 59,907 |
| Private (≥65 years old) | 2,935 (2.8%) | 1,625 (2.7%) | 427 (2.7%) | 80 (2.5%) | 156 (4.0%) | 5,223 |
| Other | 2,459 (2.3%) | 1,509 (2.5%) | 490 (3.1%) | 105 (3.3%) | 92 (2.3%) | 4,655 |
| Uninsured | 4,269 (4.0%) | 2,493 (4.1%) | 761 (4.9%) | 173 (5.4%) | 273 (7.0%) | 7,969 |
| Median Income Quartile | | | | | | |
| Highest | 28,342 (26.8%) | 18,101 (30.0%) | 3,809 (24.7%) | 714 (22.5%) | 1,027 (26.5%) | 51,993 (27.6%) |
| 2nd | 26,047 (24.6%) | 13,741 (22.8%) | 3,686 (23.9%) | 860 (27.1%) | 1,010 (26.1%) | 45,344 (24.1%) |
| 3rd | 23,174 (21.9%) | 11,936 (19.8%) | 3,719 (24.1%) | 774 (24.4%) | 707 (18.3%) | 40,310 (21.4%) |
| Lowest | 28,111 (26.6%) | 16,615 (27.5%) | 4,194 (27.2%) | 826 (26.0%) | 1,125 (29.1%) | 50,871 (27.0%) |
| Elixhauser Comorbidity Index | | | | | | |
| <0 | 29,818 (27.9%) | 17,047 (28.0%) | 4,410 (28.3%) | 820 (25.5%) | 1,117 (28.5%) | 53,212 (28.0%) |
| 0 | 47,995 (45.0%) | 27,556 (45.3%) | 6,864 (44.1%) | 1,461 (45.4%) | 1,810 (46.2%) | 85,686 (45.0%) |

© 2025 Clark NM et al. *JAMA Network Open*.

| | | | | | | |
|---|---|---|---|---|---|---|
| 1-5 | 13,233 (12.4%) | 7,326 (12.0%) | 1,801 (11.6%) | 354 (11.0%) | 446 (11.4%) | 23,160 (12.2%) |
| 6-13 | 6,177 (5.8%) | 3,427 (5.6%) | 952 (6.1%) | 201 (6.2%) | 215 (5.5%) | 10,972 (5.8%) |
| ≥14 | 9,503 (8.9%) | 5,518 (9.1%) | 1,546 (9.9%) | 381 (11.8%) | 333 (8.5%) | 17,281 (9.1%) |
| Primary Surgical Condition | | | | | | |
| Appendicitis | 16,197 (15.2%) | 9,243 (15.2%) | 2,341 (15.0%) | 468 (14.5%) | 753 (19.2%) | 29,002 (15.2%) |
| Cholecystitis | 19,861 (18.6%) | 11,402 (18.7%) | 2,889 (18.6%) | 575 (17.9%) | 735 (18.7%) | 35,462 (18.6%) |
| Hernia | 21,675 (20.3%) | 11,509 (18.9%) | 3,291 (21.1%) | 734 (22.8%) | 722 (18.4%) | 37,931 (19.9%) |
| Intestinal Obstruction | 26,872 (25.2%) | 14,997 (24.6%) | 3,956 (25.4%) | 858 (26.7%) | 997 (25.4%) | 47,680 (25.1%) |
| | | | | | | |
| Diverticulitis | 22,121 (20.7%) | 13,723 (22.5%) | 3,096 (19.9%) | 582 (18.1%) | 714 (18.2%) | 40,236 (21.1%) |
| Rurality | | | | | | |
| Metropolitan | 102,035 (95.6%) | 58,472 (96.1%) | 12,893 (82.8%) | 2,465 (76.6%) | 3,534 (90.1%) | 179,399 (94.3%) |
| Micropolitan | 3,944 (3.7%) | 1,431 (2.4%) | 1,382 (8.9%) | 303 (9.4%) | 172 (4.4%) | 7,232 (3.8%) |
| Rural | 746 (0.7%) | 971 (1.6%) | 1,298 (8.3%) | 449 (14.0%) | 215 (5.5%) | 3,679 (1.9%) |

© 2025 Clark NM et al. *JAMA Network Open*.

**eTable 2. Association between primary outcome and travel time categories, including patients who presented to a hospital outside of their home state.**

| **Covariate** | **OR** | **95% CI** | |
|---|---|---|---|
| **Sociodemographic Characteristics** | | | |
| Age (years) | | | |
| ≥80 | *Reference* | | |
| 65-79 | 0.55 | 0.51 | 0.60 |
| 40-64 | 0.77 | 0.72 | 0.82 |
| 18-39 | 1.07 | 1.02 | 1.12 |
| Sex | | | |
| Male | *Reference* | | |
| Female | 0.88 | 0.85 | 0.90 |
| Race | | | |
| Non-Hispanic White | *Reference* | | |
| Non-Hispanic Black | 0.85 | 0.81 | 0.89 |
| Hispanic | 0.85 | 0.82 | 0.88 |
| Non-Hispanic Other | 0.89 | 0.85 | 0.94 |
| Payer | | | |
| Medicaid | *Reference* | | |
| Medicare | 1.03 | 0.97 | 1.09 |
| Private (<65 years old) | 1.32 | 1.27 | 1.38 |
| Private (≥65 years old) | 1.36 | 1.28 | 1.46 |
| Other | 1.03 | 0.94 | 1.12 |
| Uninsured | 0.96 | 0.87 | 1.06 |
| Median income Quartile | | | |
| Highest | *Reference* | | |
| $2^{nd}$ | 1.01 | 0.97 | 1.04 |
| $3^{rd}$ | 1.10 | 1.05 | 1.14 |
| Lowest | 1.12 | 1.08 | 1.16 |
| | | | |
| **Clinical Characteristics** | | | |
| Elixhauser Comorbidity Index | | | |
| <0 | *Reference* | | |
| 0 | 0.38 | 0.37 | 0.39 |
| 1-5 | 0.67 | 0.64 | 0.70 |
| 6-13 | 1.19 | 1.12 | 1.26 |
| ≥14 | 1.34 | 1.27 | 1.41 |
| Primary EGS Condition | | | |
| Appendicitis | *Reference* | | |
| Cholecystitis | 0.00 | 0.00 | 0.00 |
| Hernia | 0.67 | 0.65 | 0.69 |

© 2025 Clark NM et al. *JAMA Network Open*.

| | | | |
|---|---|---|---|
| Intestinal Obstruction | 0.03 | 0.03 | 0.04 |
| Diverticulitis | 0.43 | 0.42 | 0.45 |
| **Rurality Measures** | | | |
| Rurality | | | |
| Metropolitan | *Reference* | | |
| Micropolitan | 0.94 | 0.88 | 1.00 |
| Rural | 0.83 | 0.76 | 0.91 |
| Travel Time (Minutes) | | | |
| ≤15 | *Reference* | | |
| 16-30 | 1.03 | 1.00 | 1.06 |
| 31-60 | 1.09 | 1.04 | 1.15 |
| 61-120 | 1.15 | 1.04 | 1.27 |
| >120 | 1.29 | 1.20 | 1.38 |

OR: Odds Ratio. CI: Confidence Interval. ED: Emergency Department.

© 2025 Clark NM et al. *JAMA Network Open*.

**eTable 3. Association between secondary outcomes and travel time greater than 90 minutes.**

| | Travel Time >90 minutes | | |
|---|---|---|---|
| | | | |
| **Binary Outcomes** | **OR** | **95% CI** | |
| Complex Disease at Presentation | 1.28 | 1.18 | 1.38 |
| Admission | 1.50 | 1.40 | 1.62 |
| Interfacility Transfer | 1.24 | 1.05 | 1.46 |
| Surgical Procedure | 1.00 | 0.92 | 1.09 |
| Mortality or Complications (IP only) | 1.04 | 0.89 | 1.20 |
| | | | |
| **Continuous Outcomes** | **Adjusted Difference** | **95% CI** | |
| Length of Stay (Days) | 0.53 | 0.38 | 0.67 |
| Total Charges (USD) | 7968.82 | 5041.72 | 10895.92 |

USD: United States Dollars. OR: Odds Ratio. CI: Confidence Interval. IP: Inpatient

© 2025 Clark NM et al. *JAMA Network Open*.

**eTable 4. Association between secondary outcomes and travel time greater than 120 minutes.**

| | Travel Time >120 minutes | | |
|---|---|---|---|
| | | | |
| **Binary Outcomes** | **OR** | **95% CI** | |
| Complex Disease at Presentation | 1.25 | 1.15 | 1.37 |
| Admission | 1.45 | 1.34 | 1.58 |
| Interfacility Transfer | 1.30 | 1.08 | 1.56 |
| Surgical Procedure | 0.97 | 0.88 | 1.07 |
| Mortality or Complications (IP only) | 1.04 | 0.88 | 1.23 |
| | | | |
| **Continuous Outcomes** | **Adjusted Difference** | **95% CI** | |
| Length of Stay (Days) | 0.54 | 0.37 | 0.71 |
| Total Charges (USD) | 8833.15 | 5614.72 | 12051.59 |

USD: United States Dollars. OR: Odds Ratio. CI: Confidence Interval. IP: Inpatient

© 2025 Clark NM et al. *JAMA Network Open*.
