# Supplementary material for: Travel Time as an Indicator of Poor Access to Care in Surgical Emergencies
Source: JAMA Netw Open. 2025 Jan 21;8(1):e2455258. doi: 10.1001/jamanetworkopen.2024.55258 (PMC11751744; doi:10.1001/jamanetworkopen.2024.55258)
Supplement: Supplement 2. — Data Sharing Statement [file jamanetwopen-e2455258-s002.pdf]

Data Sharing Statement

Clark. Travel Time as an Indicator of Poor Access to Care in Surgical Emergencies. *JAMA Netw Open*. Published January 21, 2025. doi:10.1001/jamanetworkopen.2024.55258

Data
Data available: No

Additional Information
Explanation for why data not available: HCUP is a data set with restricted access and all researchers who interact with the data are required to complete a Data Use Agreement. Unfortunately, this restriction includes journal readership access according to our reading of HCUP restrictions.
